# A New American University Model for Training the Future MCH Workforce Through a Translational Research Team

**DOI:** 10.1007/s10995-021-03349-3

**Published:** 2022-01-20

**Authors:** Corrie M. Whisner, Jean C. Brown, David M. Larson, Lizeth Alonso Rodriguez, Beate Peter, Elizabeth Reifsnider, Jennie Bever, Li Liu, Erin Raczynski, Jose-Benito Rosales Chavez, Chinedum Ojinnaka, Cady Berkel, Meg Bruening

**Affiliations:** 1 College of Health Solutions, Arizona State University, 500 N. 3rd St, Phoenix, AZ 85004 USA; 2 REACH Institute, Arizona State University, 900 S. McAllister Ave, Tempe, AZ 85287 USA; 3 Edson College of Nursing and Health Innovation, Arizona State University, 550 N. 5th St, Phoenix, AZ 85004 USA; 4 4th Trimester, Arizona 8400 S Kyrene Rd., Suite 126, Tempe, AZ 85284 USA; 5 Arizona Department of Education, 1535 W Jefferson St, Phoenix, AZ 85007 USA; 6 School of Geographical Sciences and Urban Planning, Arizona State University, 975 S Myrtle Ave., Tempe, AZ 85281 USA

**Keywords:** Training, Workforce, Maternal, Child, Translational research

## Abstract

**Objectives:**

To describe the process of developing and implementing experiential learning through translational research teams that engage diverse undergraduate and graduate students.

**Methods:**

After a college redesign, translational research teams were developed to foster multidisciplinary research and better integrate students with faculty research, community, and clinical activities. Three primary approaches were used to engage undergraduate and graduate students in the maternal and child health translational research team (MCH TrT). These included an undergraduate experiential learning course; participation in translational research team meetings and events; and mentorship activities including graduate student theses and supplementary projects.

**Results:**

Since 2019, a total of 56 students have engaged with the MCH translational research team. The majority (64%) of students engaging in translational research were undergraduates. Racial and ethnic diversity was evident with 16% Latinx, 14% Black/African American, 12% Asian, 10% two or more races, and 4% Native American or Native Hawaiian. A large proportion (42%) of students indicated that they were first-generation college students, while 24% indicated they had a disability. Five themes emerged from student feedback about their involvement in the experiential learning course: the value of translational research, development of research skills, collaboration, practice development, and value for community partners.

**Conclusions for Practice:**

Through an MCH translational research team, we have established a pathway to enhance diversity among the MCH workforce which will increase recruitment and retention of underrepresented groups, and ultimately improve MCH research and practice.

## Significance

The literature is rich on the importance of engaging students in translational science with suggestions for models on how to implement this work; however, very little research has described the actual implementation of translational research teams, particularly in maternal and child health. We established a robust maternal and child health translational research team intending to engage both undergraduate and graduate students in multidisciplinary team science. Data collected since establishing the team in 2019 suggest that bringing together a diverse group (expertise, employer, career stage, and race/ethnicity) of passionate researchers, MCH and practitioners supports student growth and engagement.

## Introduction

The MCH workforce is well-educated, highly motivated, and skilled in evidence-based practice and communication, but the current health workforce is not fully prepared to serve a fast-changing population (Rhea & Bettles, 2012), particularly in the southwest, which includes the highest populations of Native Americans and Latinos in the United States. This is especially true, given that cultural matching of health professionals and clients is known to reduce mistrust and improve communication and health outcomes (Wakefield, 2014). According to HRSA’s report on *Sex, Race, and Ethnic Diversity of U.S. Health Occupations (2011–2015)*, Latinos and Black /African Americans are significantly underrepresented in nearly all MCH occupations, and Native Americans make up only 1% of the MCH workforce (U.S. Department of Health and Human Services 2017).

MCH is a dynamic field that requires evolving training mechanisms for future MCH workers. From their 2016 survey, the Association of Maternal and Child Health Programs (AMCHP) has identified nine current and future workforce needs and gaps in skills that educators should begin to cover in public health and related curricula (“AMCHP Workforce Development Survey” 2016). These workforce needs include public health and Title V knowledge base, communication, critical thinking, management skills, family-centered care, and medical home training, evidence-based decision making, leadership skill development, change management, and systems thinking and integration. Efforts are needed to expose students to this content to fill these gaps before joining the workforce. Furthermore, these efforts should be administered with diversity, equity, and inclusion at the core of training by continually emphasizing cultural competency for MCH health and welfare.

To sustainably meet growing MCH workforce needs, universities may need to identify and engage students from non-traditional MCH academic programs. This is especially true for institutions that do not have structured MCH departments and/or academic programs. This paper describes the process of developing and implementing those activities for undergraduate and graduate students at a large southwestern university with non-traditional MCH academic programs. After a college redesign, translational research teams, including the MCH Translational Research Team (MCH TrT), were developed to foster multidisciplinary research and better integrate students with faculty research, community, and clinical activities. In the methods section, we describe the structure of the MCH TrT and opportunities for students. In the results, we present descriptive and qualitative feedback from students about their involvement. Findings will apply to engaging students on interdisciplinary research within and outside of traditional MCH learning environments to promote a diverse workforce and long-term retention in the field of MCH.

## Methods

### Translational Research as an MCH Training Model

Several innovations have occurred at Arizona State University (ASU), the largest university in Arizona and one of the 10 largest universities in the United States, which have facilitated unique opportunities for students interested in the MCH field. At the university level, ASU established its charter as the New American University with eight design aspirations:

1. Leverage Our Place: ASU embraces its culture, socioeconomic and physical setting.
2. Enable Student Success: ASU is committed to the success of each unique student.
3. Transform Society: ASU catalyzes social change by being connected to social needs.
4. Fuse Intellectual Disciplines: ASU creates knowledge by transcending academic disciplines.
5. Value Entrepreneurship: ASU uses its knowledge and encourages innovation.
6. Be Socially Embedded: ASU connects with communities through mutually beneficial partnerships.
7. Conduct Use-Inspired Research: ASU research has purpose and impact.
8. Engage Globally: ASU engages with people and issues locally, nationally, and internationally.

Many of these design aspirations align with MCH Leadership Competencies (U.S. Department of Health and Human Services, Health Resources and Services Administration, Maternal and Child Health Bureau 2018) and allow faculty to more easily collaborate across disciplines to reach underrepresented students and do important work with MCH communities. Inspired by the 8 university-level design aspirations, the College of Health Solutions (CHS) made two important changes relevant to MCH students. First, the college was reorganized to support transdisciplinary, translational research and education to accelerate discovery and impact on health and health disparities. We moved from traditional departments and programs to translational research teams (focused on populations or health conditions) and affinity networks (focused on methodology) developed by members of the faculty. This new “flat” structure is designed to promote crosstalk and interprofessional teaching and research, ideal for training the next generation for the workforce. Translational research teams bring together interdisciplinary faculty, students, and community partners to develop innovative research and practice solutions focused on Maternal Child Health, Metabolic Health, Autism Spectrum Disorder, Language and Literacy, COVID-19, Cleft Palate, and Oral Health. The MCH TrT aims to create a community of practice and a collaborative network for transdisciplinary research across the translational spectrum. As a grassroots structure, the activities of the MCH TrT are driven by the interests and expertise of its members. Second, all undergraduate students in the CHS are now required to obtain three credits of experiential learning before graduation. Our MCH TrT developed several opportunities to provide experiential learning opportunities to engage students in MCH research and practice.

### MCH Translational Research Team Organization and Activities

**Fig. 1:**
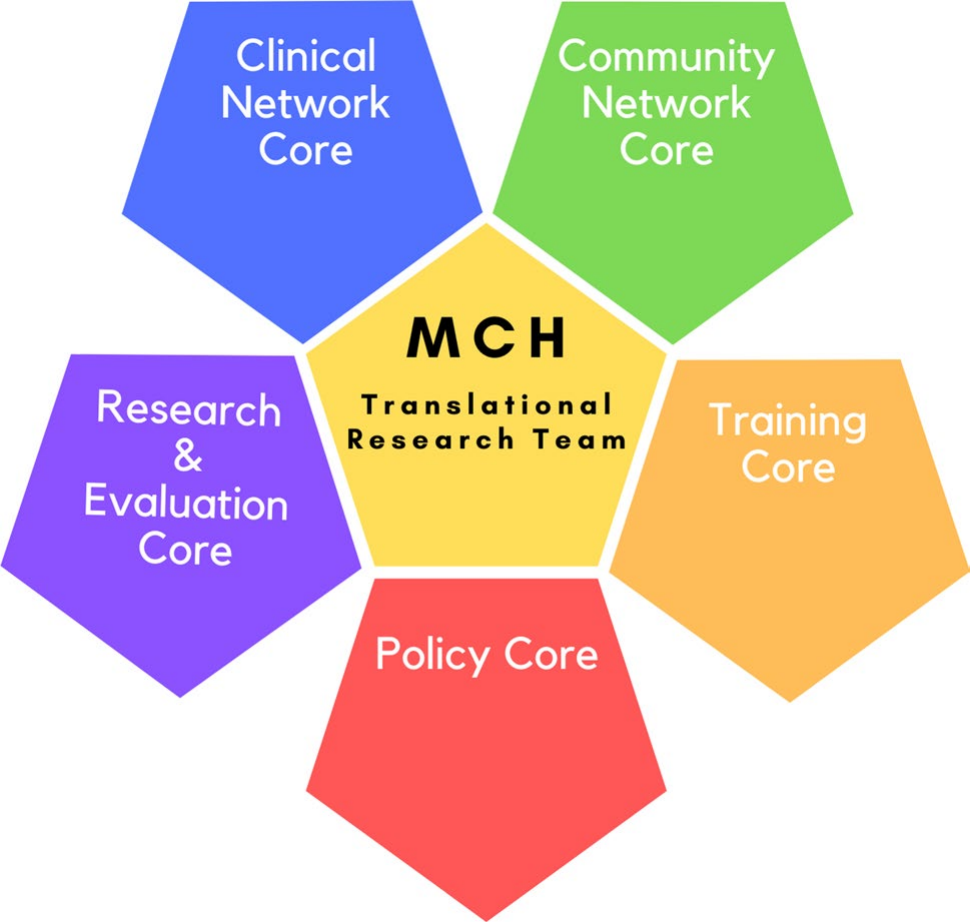
Organizational structure of the Maternal Child Health Translational Research Team (MCH TrT)

**Fig. 2:**
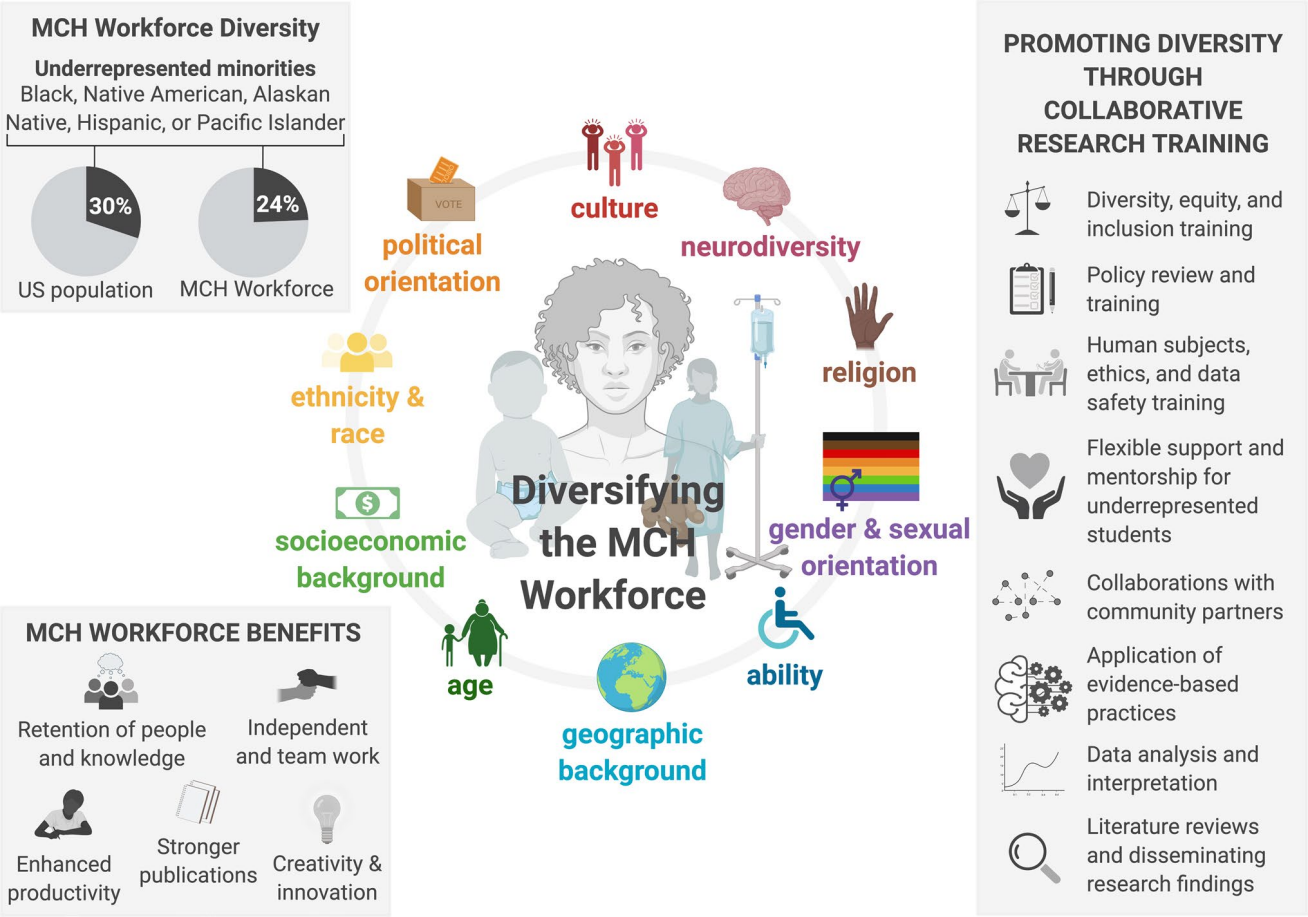
A summary of the New American University model for research engagement and career development training for the future MCH workforce

The organizational structure of the MCH TrT includes five cores (teaching, research, community partnerships, healthcare partnerships, and policy) that support team projects (see Fig. 1). All of the research projects include at least one faculty member, one community partner, and one student. This structure provides diverse opportunities for student learning, as evidenced by current and past projects (see Fig. 2**)**. These opportunities primarily fit into one of the following categories: (1) participation in overall MCH team meetings and events, (2) enrollment in an online course designed to prepare undergraduate students for community-based research, and (3) involvement in team-based research projects. Students were provided with a variety of experiential opportunities as part of the MCH TrT. Examples of these activities were cross-walked with the AMCHP workforce development needs to demonstrate illustrative activities that fill those gaps in student training (see Table 1). More details about team engagement, student training, and research project involvement are presented in greater detail below.

**Table 1:** Selected examples of student experiential learning activities in MCH research projects as they apply to workforce development needs and resulting project outcomes

AMCHP Workforce development needs^a^	Translational team project/activity	Student engagement activities	Project outcomes
*Public health and title V knowledge base*: Translating data into viable information	Assessing the potential of telehealth support to engage and support diverse families affected by Neonatal Abstinence Syndrome and other life-limiting conditions	Literature reviews; Human subjects; Database development & data cleaning; Data analysis	Infographic on best practices for telehealth delivery; Capstone Project; Funding for continued data collection and analysis
*Communication*: Delivering difficult or sensitive health status information	Opioid use among pregnant women	Literature reviews	Summary of health outcomes related to perinatal opioid use
*Critical thinking*: Skills in systems thinking, identifying whole situations	Interactions among genetic variations, brain structures and functions, biomarkers, and communication behaviors for at-risk children	Participant recruitment; quality control of the clinical implementation; phonetic transcription of babble and early words	Recruitment and implementation of a complex intervention with over 50 families
*Management skills*: Knowledge and skills to nurture a positive organizational culture	Monthly translational team meetings and breakout sessions	Collaborative team visioning activities; Brainstorming sessions involving researchers, community partners and students	Established strategic plan for next 3–5 years; Created SMART goals and identified deliverables for team cores
*Family-centered care and medical hom***e**: Solicit family partnerships in a meaningful way in the design, delivery and services and programs	Care coordination for children with complex health care needs	Implementation of care coordinator between acute care and outpatient units	Student project and publication
*Evidence-based decision making*: Critically examine evidence-based interventions against identified needs	Child and adult care food program (CACFP): Exploring differences in providers who participate vs. who do not and what barriers eligible sites experience	Data procurement, analyses, and dissemination; engagement of cross-agency partners	Thesis; new outreach plan to increase CACFP sites; preliminary data for NIH grant (now funded)
*Development of leadership skills*: Knowledge and skills to collaborate with partners	Evaluating the effects of sleep–wake patterns and gut microbiome development on infant growth	Student teams organized to have leadership over specific duties/tasks (e.g. Relationship building with clinical partners and community organizations)	Generated list of potential contacts; Developed communication materials and tools for partners to facilitate collaboration
*Change management*: Lead teams to maximize buy-in, keep members engaged, and sustain efforts over time	COVID analytics for MCH populations	Manage constant changing landscape regarding the collection of COVID-related data	Novel interdisciplinary translational partnerships;
*Systems thinking and integration*: Use systems mapping methods to understand current systems	Integration of family-based prevention in integrated primary care settings	Preparing materials for assessments and program implementation; Contact with families; Data entry; Payor data collection	Evidence to support the integration of family-based prevention in primary care to address social determinants of health and improve behavioral and physical health equity

^a^Based on AMCHP training needs identified in the 2016 workforce development survey

### Participation in Overall MCH Team Meetings and Events

MCH TrT meetings are held monthly. All members, including students, are invited to participate. Students have an equal voice in all activities. Meeting agendas include information-sharing sessions for community partners, researchers, and students, and identification of new projects/needs for existing projects. In addition, meetings are used for collaborative strategic planning activities, in which breakout sessions are held for each of the five cores to develop and implement goals to support the team. Students are active participants in this planning process. In addition to our monthly meetings, we also hold an annual MCH conference in which students can present their research findings to the broader MCH community across the state.

### Undergraduate Student Activities—Community-based Research Course and Research Projects

Each semester, undergraduate students can enroll in our MCH TrT 3-credit course (135 h), which fulfills their experiential learning requirement in the CHS. The course is designed to provide the background for participation in team research projects (see Table 1). Given the diversity of research projects, students enrolled in the course are required to complete six self-paced modules (Table 2) before engaging in research. This ensures that a core set of knowledge and skills are shared across all student trainees and that all trainees have training in research with human subjects. Research teams create short presentations that explain the project and opportunities for student involvement. Students reach out to several potential research mentors to identify a match. Once the student and research/mentor matches are completed, students and their research mentors agree upon activities and expectations.

**Table 2:** Learning modules in the undergraduate experiential research course

Module	Learning objectives
Translational research	Describe levels of integration within team science Compare and contrast the five stages of translational science
Importance of MCH	Discuss the life course approach List critical areas in MCH
Ethics, equity, and evidence-based practice	List the seven steps to evidence-based practice Discuss the importance of health equity
Interprofessional skills	Identify roles and responsibilities on research teams Describe tips for improved interprofessional communication Discuss components of strong teams and teamwork
Research ethics	Describe the rationale for protecting human subjects in research
Policy	Analyze the policy process Discuss the role of policy in MCH Compare strategies for becoming advocates for MCH

### Graduate Student Activitie—Research Engagement and Leadership Experience

Like the undergraduate students, graduate students are assigned to team projects; however, they have the opportunity to take on more leadership roles in the research. For example, some students have engaged in projects as part of their thesis work, resulting in leading data collection, analyses, and dissemination. Other students take on roles supplementary to their research to gain additional research skills and contribute to interdisciplinary expertise. An example of a supplemental project is engaging in data analysis and paper-writing sprints to assist community and state organizations with health programming and resource allocation. Almost all graduate students participate in mentorship roles, providing leadership and teaching to undergraduate students on their respective teams.

### Study Design and Participants

We surveyed a convenience sample of students participating in the MCH TrT, either voluntarily or for course credit. Students who volunteered or enrolled in the MCH TrT course were recruited through digital academic advising newsletters, flyers, and word of mouth. Both graduate and undergraduate students were targeted by these recruitment methods. To evaluate student experiences, we reviewed student writing assignments and course evaluations to identify common themes. As a group, we identified passages that related to students’ perceptions of their learning and development. To obtain community partner perspectives about working with students, we reviewed written qualitative feedback from MCH TrT members collected by an external evaluator as part of our strategic planning process. We categorized these written responses into categories and then identified representative quotes for each.

Approximately $${\raise0.7ex\hbox{$2$} \!\mathord{\left/ {\vphantom {2 2}}\right.\kern-\nulldelimiterspace} \!\lower0.7ex\hbox{$2$}}$$ of students affiliated with the MCH TrT were pursuing undergraduate degrees and $${\raise0.7ex\hbox{$1$} \!\mathord{\left/ {\vphantom {1 2}}\right.\kern-\nulldelimiterspace} \!\lower0.7ex\hbox{$2$}}$$ were pursuing graduate degrees. Student majors include, but are not limited to biology, biomedical engineering, medical studies, nutrition, neuroscience, nursing, public health, psychology, and social work. The CHS is highly diverse (Table 3); its racial/ethnic minority membership slightly exceeds that of the university as a whole (48%). Student diversity of the MCH TrT surpassed the college’s numbers with 61% racial/ethnic minority. The majority of students identified as cisgender female (76%), the rest of the students identified as cisgender male (16%) or preferred not to identify (8%); additionally, 8% identified as gay, lesbian, or bisexual. A large proportion (42%) of students indicated that they were first-generation college students; 24% indicated they had a disability.

**Table 3:** Student demographics

	College of Health Solutions	Undergraduate	Graduate	MCH TrT
Latinx	25%	28%	14%	16%
Black/African American	7%	7%	11%	8%
Native American	2%	2%	2%	2%
Asian	6%	6%	7%	12%
Native Hawaiian/Other Pacific Islander	< 1%	< 1%	1%	2%
Middle Eastern/North African	NA	NA	NA	6%
Non-Latino White	51%	51%	53%	40%
Two or More Races	5%	5%	4%	10%
Not Available	1%	1%	2%	4%

*MCH TrT* Maternal and Child Health Translational Research Team

## Results

The themes identified included the value of translational research, development of research skills, effective collaboration, and changing their practice with MCH populations based on the content of what they studied. Across all themes, the responses were positive; the only negative comment received was concerning a potential faculty mentor not responding to student inquiries about possible research mentorship promptly.

### Value of Translational Research

Students showed growth in their understanding of the importance of translational research. One student said: “My work showed me how translational research acts as the backbone for any subject since it does not only research a topic but helps with providing possible methods to create better health outcomes.” Another student reflects on the life-long learning that comes with translational research: “I think that research in general is never ending. Just when you find answers, another question presents itself. I think that is just how life is, find something, but look some more. Learning should never end.”

### 3.2 Research Skill Development

Students appreciated learning new skills and could see how it would impact their long-term career goals. One student noted the following in their end-of-course reflection assignment: “I now know how to conduct research and literature reviews, I now know how to use translational research to improve health outcomes and I now know how to collaborate with a research team. I developed many skills that I will use in the future as I conduct more research and skills that I can use while pursuing a career in healthcare.”

### Effective Collaboration

Students described how the interdisciplinary team experience impacted how they interact with others and how they understood their role in the process. One noted, “The more mature I become, the more I realize the importance of a team. I learned this being a part of this massive research team and learned every moving piece of the puzzle is important no matter how big or small. From the people who coordinate the projects, to those who execute the ideas, to the supporting roles, and even to the billing, everyone has something to contribute.”

### Practice Development

Students indicated how they will change their practice approaches in their respective areas of MCH as a result of the experiential learning. A student who worked on a project related to Neonatal Abstinence Syndrome wrote: “I plan on thinking more about prenatal exposure to a variety of substances when working with clients who may suffer from disease or mental health disorders. Asking questions about their mother’s habits when they were pregnant can be helpful in understanding some influences on current determinants of health.”

### Value for Community Partners

Community partners benefitted from student involvement in terms of the research skills and time students were able to contribute. For example, one community partner proposed a needs assessment as a project for the MCH TrT. A student took this on as a Master’s thesis. As a result of the thesis, the community partner has changed their practice. Moreover, the results were used as preliminary data for a grant that was funded. The community partner noted: “I have been so grateful… The needs assessment that I didn't have the capacity to do on my own and the work of this team helped me make informed decisions as a practitioner and a program administrator so that I could better serve my community. I am so grateful for the work that we did in collaboration with a student … I've relied on it; I can't tell you how many times I’ve pulled the data over the last like six to eight weeks. So the value I don't think I could understand it or overstate it and, like it has served me immensely as a community partner.”

## Discussion

Translational research teams can seamlessly integrate learning, community outreach, and research to train the future MCH workforce and could lead to better retention. By leveraging externally funded research and novel interdisciplinary projects as a result of new connections among the MCH TrT, we have created formal (courses and training grants) and informal systems (team activities, volunteering) to engage graduate and undergraduate students in interdisciplinary MCH research, outside of a traditional MCH department. While the literature is rich on the importance of engaging students in translational science, and suggestions for models exist on how to implement this work (Brunson & Baker, 2016; Calhoun et al., 2013; Hobin et al., 2012; Neuhauser et al., 2007), very little research has described the actual implementation (Haynes et al., 2020). We demonstrate that translational research teams can be successful in engaging undergraduate and graduate students in multidisciplinary team science through a formal college-wide translational research team. The model of the university allows the flexibility for a translational research team to thrive and bring together a diverse group (in expertise, place of employment, stage of career, and demographics) of passionate researchers, practitioners, and advocates committed to making an impact for MCH populations in Arizona (2019) while supporting student growth and engagement. Given that most non-MCH program curricula are focused on chronic disease prevention, our model is an exemplar in practice that others could adapt and test to build the MCH workforce while impacting populations in need. Without having a formal program in MCH, we have shown that interdisciplinary research teams expand the exposure of MCH to students. Further, this model could be used by existing MCH-programs to enhance collaborations and create communities of practice and pathway programs for MCH program graduates.

Experiential learning creates the opportunity for flexibility to create transferable skills so that students apply evidence-based skill sets to use in their future careers (Kolb & Kolb, 2018; Morris, 2020). Students have indicated that as a result of their engagement in the translational research team activities, they have enhanced their skills in the use of research evidence, practice development, communication and team building, and research, all components of gaps identified in the AMCHP workforce development survey (“AMCHP Workforce Development Survey” 2016). Previous evidence corroborates this finding. Among liberal arts schools, STEM undergraduate experiential learning programs led to greater facilitation of career goal development, the evolution of collaborative teamwork skills, and ownership of real-world projects (Thiry et al., 2011). In other studies, students identified the greatest benefits of research and professional experience internships as personal/professional gains, increased application of knowledge and skills, and advancement of skills (Seymour et al., 2004) which mentors then linked to professional growth and preparation for careers in science (Hunter et al., 2007).

How engagement in experiential learning is related to long-term career choices and retention within the MCH field remains largely unstudied. With just 10% of the MCH workforce below the age of 30 years (AMCHP Workforce Development Survey 2016), it is clear that current training falls short for placing and retaining qualified graduates. Innovative workforce development solutions like the MCH TrT proposed here may help to train more MCH professionals while exposing them to authentic MCH projects and experiences. Successful implementation of experiential learning among life sciences graduate students and postdoctoral trainees has been shown to help participants make informed career decisions through exposure to various professions and job cultures, transferrable skills development, and evaluation of personal skill sets and strengths for specific careers (Schnoes et al., 2018). Although our work as a translational research team is in an early stage, the response from students regarding the application of knowledge and skills, and practice development alongside community organizations and partners suggest that trainees may have a clearer picture of the MCH workforce.

Interprofessionalism is a key strength to translational research teams and has been shown to effectively prepare health workers for collaborative projects, leadership, and improved patient care (van Diggele et al., 2020). The translational team provides a model for students on how to work in integrated and collaborative public health and healthcare environments. All of the research projects engaged at least one faculty member, one community partner, and one student. Team meetings discussed complex, cross-cutting issues such as the opioid epidemic in pregnant women, working on coordination of care for children with special health care needs, or adapting practice in the heart of the COVID-19 pandemic, as a few examples. The students observed and participated with professionals from multiple areas of expertise as they organically identified, planned, and collaborated on projects. Future research is needed on how these integrated activities enhance student training outcomes, and MCH career recruitment and retention.

A safe and supportive learning environment is especially critical for students from underrepresented groups. Despite the diversity of our student body, only 14% of the college faculty are from underrepresented racial/ethnic backgrounds, which increases the likelihood of students’ confronting bias or microaggressions (Harper, 2013; Smith et al., 2007), which have a direct effect on the potential for a diverse workforce (Hurtado et al., 2015). This is a challenge we are currently addressing through several initiatives, including activities of our Justice, Equity, Diversity, and Inclusion (JEDI) council.

While these efforts will take time to transform the college faculty, participation in the MCH TrT may help students to find a sense of belonging. Previous research suggests that mentorship for culturally and linguistically underrepresented students has many benefits including enhanced learning through practical experiences (Hunter et al., 2007), greater development of research-related skills and independence (Haeger & Fresquez, 2016), and a greater likelihood of staying in their field of choice and achieving career goals (Schultz et al., 2011). Translational research teams that integrate faculty and student mentorship with the community and clinical partnerships offer a robust and diverse mentoring program, as evidenced by student feedback. The higher rates of student diversity in the MCH TrT compared to the college or university may suggest that underrepresented students may be attracted to conducting meaningful research with diverse MCH populations. In addition, the diversity of the faculty may be supplemented by the diversity of community partners as well as other students, particularly when more senior students are available to mentor their junior colleagues.

Finally, training on diversity, equity, and inclusion is a well-received staple of our annual conferences, and we will continue to identify additional ways to weave it into the fabric of the MCH TrT. We hope that our demonstrated ability to attract a diverse cadre of students will help us to increase the diversity of our faculty, with bidirectional effects leading to a MCH workforce that more adequately represents the population it serves.

Despite our current positive feedback for the MCH TrT as a mechanism to train future MCH workers, there is room for expansion. A limitation of our current training mechanism is that experiential learning is heavily tied to the match with current faculty and community partner interests and/or availability of funding to support research projects. Authentic and productive community-based research projects can take a long time to solidify, and even longer to obtain funding, and produce data for students. As a result, a topic of interest to a student might not necessarily be ready to work on at any given time. For example, community partners have recently approached us to launch projects related to adolescent reproductive health, community-based prenatal and postpartum support for Black mothers, the benefits of cultural health navigators for refugee women’s health, and nutrition for young children, yet these will each take time to be ready for tangible student projects.

## Conclusion

We have recognized that robust educational opportunities are needed to adequately train a diverse workforce who is prepared to lead in the field of MCH. Educational strategies have to be accessible, innovative, meaningful, and tailored to the needs of the workforce. Training a diverse body of undergraduate and graduate students that reflect the demographics of Arizona (2019) and the broader Southwest will encourage greater creativity, promote productivity, and result in teaching and research products that better equip a diverse workforce in MCH. Through the translational research team, we have also established a pathway to enhance diversity among the MCH workforce which will ultimately improve workplace culture, enhance recruitment and hiring practices, increase retention, and potentially improve MCH.

## Data Availability

Interested parties can request access to de-identified data by contacting the Corresponding Author.
